# Cell death paradigms in the pathogenesis of *Mycobacterium tuberculosis* infection

**DOI:** 10.3389/fcimb.2014.00031

**Published:** 2014-03-05

**Authors:** Dinesh Kumar Parandhaman, Sujatha Narayanan

**Affiliations:** ^1^Department of Immunology, National Institute for Research in TuberculosisChennai, India; ^2^Department of Immunology, International Centre for Genetic Engineering and BiotechnologyNew Delhi, India

**Keywords:** apoptosis, pyroptosis, intrinsic pathway, extrinsic pathway, autophagy, *Mycobacterium tuberculosis*

## Abstract

Cell death or senescence is a fundamental event that helps maintain cellular homeostasis, shapes the growth of organism, and provides protective immunity against invading pathogens. Decreased or increased cell death is detrimental both in infectious and non-infectious diseases. Cell death is executed both by regulated enzymic reactions and non-enzymic sudden collapse. In this brief review we have tried to summarize various cell death modalities and their impact on the pathogenesis of *Mycobacterium tuberculosis*.

## Introduction

Cell death is a primordial event in embryogenesis, metamorphosis, and in innate immune response against the invading pathogens. Cell death as a defense mechanism is also documented in the plant kingdom (Kabbage et al., 2013). Cell death is executed in a series of ordered biochemical cascades and is referred as programmed cell death or PCD.

Till early 2000, cell death was discussed as dichotomy in terms of either apoptosis or necrosis. However, with the growth of science many distinct modes of cell death with well-organized signaling cascades were unraveled. Currently, there exists nine different forms of cell death namely apoptosis (Fink and Cookson, 2005), autophagy (Fink and Cookson, 2005), mitoptosis (Chaabane et al., 2012), necrosis (Fink and Cookson, 2005), necroptosis (Galluzzi and Kroemer, 2008), netosis (Remijsen et al., 2011), oncosis (Fink and Cookson, 2005), pyroptosis (Fink and Cookson, 2005), and pyronecrosis (Willingham et al., 2007). It is still a puzzle whether these pathways are different features of the same response or physiologically distinct responses. Apoptosis as an defense mechanism initiates both innate and adaptive immunity (Behar et al., 2010). However, pathogenic organisms have developed mechanisms to modulate apoptosis for their survival. Apoptosis of the infected cells have been reported to be a favorable outcome for the dissemination of infections like Yersinia, Francisella, etc. (Ruckdeschel et al., 1997; Wickstrum et al., 2009). On the contrary, impairment of apoptosis provides a survival niche to many intracellular pathogens including *Mycobacterium tuberculosis* (Behar et al., 2010), leads to auto immunity, cancer and degenerative disorders (Elmore, 2007). Studies in *M. tuberculosis* have identified a causal relationship between virulence of the strain and induction of apoptosis. Inhibition of apoptosis favors *M. tuberculosis* survival in many ways like preventing bactericidal effects, T-cell priming, etc. (Velmurugan et al., 2007). In contrast, a recent report states that apoptosis inducing strains could disseminate *M. tuberculosis* infection (Aguilo et al., 2013). Necrotic cell death of burdened *M. tuberculosis* infected cells was shown to pave way for re-infection (Butler et al., 2012). In here, we summarize various apoptotic modalities and their role in the pathogenesis of *M. tuberculosis*. Furthermore, we share our experience in analyzing these responses in *M. tuberculosis* infection.

## Models of cell death

### Apoptosis

First represented in the article by Kerr, Wyllie, and Currie in 1972 (Elmore, 2007). Apoptosis is an energy dependent regulatory process that disintegrates the dying cell by enclosing the cytoplasmic contents inside membrane bound vesicles called apoptotic bodies. These apoptotic bodies are engulfed by the phagocytic cells by a process called efferocytosis thereby efficiently clearing the dying cell without any inflammatory responses (Lee et al., 2009). Three pathways namely extrinsic/ligand-mediated pathway, intrinsic/mitochondrial pathway, and the granzyme B-mediated pathway regulate the process of apoptosis upon activation by physiological or pathological conditions (Elmore, 2007). The major players in apoptosis are caspases, adaptor proteins, tumor necrosis factor (TNF) receptor (TNF-R) super family, and Bcl-2 family of proteins (Strasser et al., 2000). There are three categories of caspases; initiators (caspase-2,-8,-9,-10), effectors or executioners (caspase-3,-6,-7), and inflammatory caspases (caspase-1,-4,-5) (Elmore, 2007). Caspase-activated DNases activate endonuclease that produce the typical internucleosomal DNA cleavage during apoptosis (Strasser et al., 2000). Adapter proteins play a major role in apoptosis as a link between caspases and the TNF-R by mediating homotypic interactions between the domains death domain, the death effector domain, and the caspase recruitment domains (Strasser et al., 2000).

Bcl-2 family of proteins are classified into three types that fall into pro-survival and pro-apoptotic categories based on the amino acid sequence homology to Bcl-2 homology regions BH1–BH4. Pro -survival Bcl-xL, Bcl-w, A1/Bfl-1, Mcl-1, and Boo/Diva have three or four bcl-2 homology regions while the pro-apoptotic members called Bax-like death factors Bax, Bcl-xS, Bak, and Bok/Mtd contain two or three homology regions (Pecina-Slaus, 2010). The third group of proteins Bad, Bik/Nbk, Bid, Hrk/DP5, Bim/Bod, and Blk, etc. that possess only a BH3 region are potent inducers of apoptosis (Strasser et al., 2000).

#### Apoptotic pathways

- **Extrinsic pathway** is initiated by binding of the ligands like TNF-α, FasL, CD95L, TRAIL, etc. to their respective receptors TNFR, Fas/CD95, and DR3 on the cell surface. This activates the initiator caspases such as caspases 8 and 10 that results in the formation and activation of death inducing signaling complex (DISC) that activates caspase 3 (Pecina-Slaus, 2010; Kalimuthu and Se-Kwon, 2013). Caspase 3 activation leads to cleavage of various death substrates that results in the characteristic hallmarks of apoptosis like DNA fragmentation, membrane blebbing, etc. (Kalimuthu and Se-Kwon, 2013).
- **Intrinsic pathway** of apoptosis is trigged due to the intracellular death signals. Mitochondrial enzyme endonuclease G, Bcl-2 family of proteins like Bax, Bid, and other mitochondrial proteins AIF, DIABLO [SMAC (second mitochondria-derived activator of caspases)], and cytochrome C plays a major role in this response (Kalimuthu and Se-Kwon, 2013). Upon the stimulus, the BH3-only protein Bid activates Bax and Bak that results in conformational change and oligomerization, forming an oligomeric pore in the outer mitochondrial membrane called permeability transition pores (Ferri and Kroemer, 2001; Kalimuthu and Se-Kwon, 2013). This results in the release of cytochrome C and other pro-apoptotic factors from the mitochondria into the cytosol. Cytochrome C interacts with Apaf and activates caspase-9 forming a multi-protein subunit complex called casposome (apoptosome) comprising cytochrome C, Apaf-1, procaspase-9, and ATP. In the absence of death stimulus, inhibitor of apoptosis family proteins (IAP) inactivates the caspase activity by direct binding. However, upon apoptotic stimuli IAPs are negatively regulated by SMAC and that leads to the activation of caspase-3 (Pecina-Slaus, 2010; Kalimuthu and Se-Kwon, 2013). Furthermore, extrinsic pathway was found to influence the intrinsic pathway of apoptosis by truncation of Bid (Cillessen et al., 2007).
- **Granzyme B**-mediated pathway utilizing the extrinsic mode of apoptosis is used by cytotoxic T lymphocytes as a mechanism to kill its target. Besides this, the secretion of pore forming granules containing serine proteases granzyme A and granzyme B also execute apoptosis that is both dependent and independent of caspase activation (Elmore, 2007).

### Autophagy

It is a regulated homeostatic response conserved in all living cells degrading their own cytoplasm. Autophagy is a predominant cell survival response that is involved either in nutrient turnover or energy production during stress or removal of long lived cells or to protect against invading intracellular pathogens (Chaabane et al., 2012). Three forms of autophagy namely macroautophagy, microautophagy, and chaperone-mediated autophagy exist. During the autophagy, damaged organelle is lined with an isolation membrane called the phagophore that enlarges forming the double membrane structure called autophagosome. The autophagosome fuses either with late endosomes or lysosomes causing cell death (Levine and Deretic, 2007; Remijsen et al., 2011). Autophagy is regulated by autophagy-related proteins, serine/threonine kinase, mammalian target of rapamycin (mTOR), class I and class III phosphoinositide 3-kinases (PI3Ks) (Levine and Deretic, 2007; Su et al., 2013).

### Mitoptosis

Apoptotic changes inside the mitochondria are called mitoptosis. Mitoptosis is still in infancy and no specific factors have been identified. The identification is based on morphological changes like disintegrating cristae, swollen mitochondria, etc. (Chaabane et al., 2012).

### Necrosis

Accidental cell death induced due to pathological or physiological conditions are called necrosis. During necrosis, swelling of organelles like endoplasmic reticulum, mitochondria occurs thereby rupturing the plasma membrane. This leaks the intracellular contents of the necrotic cell into the intercellular space causing inflammatory responses (Fink and Cookson, 2005; Chaabane et al., 2012).

### Necroptosis

In the year 2008, Hitomi et al. reported that necrosis could be a regulated process of cell death. The activation of serine/threonine kinase RIP1, BH3 only protein Bmf, and mitochondrial dysfunction executes necroptosis (Galluzzi and Kroemer, 2008).

### NETosis

In 2004, the findings of Brinkman group unveiled another cell death program named by Steinberg in 2007 called NETosis (Mesa and Vasquez, 2013). One among the defense mechanisms used by neutrophils is the extrusion of intracellular material in the form of extracellular traps (ETs) to the surrounding extracellular medium. This concentrates the microbicidal substances to trap and kill pathogens (Mesa and Vasquez, 2013). Release of ETs by neutrophils is called NETs and mast cells as MCETs. NETs are composed of DNA and histones, and they are resistant to degradation by proteases, insensitive to caspase inhibition and necrostatins (cytoprotective agents) (Mesa and Vasquez, 2013). During NETosis both the nuclear and granular membranes disintegrate leaving the plasma membrane intact (Remijsen et al., 2011). NETosis is activated by pathogens, platelets activated with LPS and in eosinophils (Remijsen et al., 2011). Formation of NET is both nuclear and mitochondrial in origin.

### Oncosis

It is the swelling of cells that involves rapid plasma membrane breakdown, and swollen nuclei without internucleosomal DNA fragmentation. Oncosis depletes cellular energy and leads to failure of the ionic pumps in the plasma membrane. It is elicited by agents that disrupt the ATP production of the cell (Fink and Cookson, 2005).

### Pyroptosis

Apoptosis in general does not induce an inflammatory response. However, apoptosis in Shigella, Salmonella, Francisella, and Legionella infections produce inflammatory responses that are called as pyroptosis (Carneiro et al., 2009; Lee et al., 2011). Pyroptosis is executed by the formation of inflammasomes by bacterial products involving NLRC 4 (Nod-like receptor—NLR), that activates caspase-1 and the processing of IL-1β and IL-18 cytokines promoting cell death (Fink and Cookson, 2005; Carneiro et al., 2009).

### Pyronecrosis

Cathepsin B-dependent apoptosis that is independent of caspase-1 activation and inflammasome formation is called pyronecrosis. This mode of apoptosis is observed in shigellosis (Willingham et al., 2007; Carneiro et al., 2009).

### Other apoptotic models

- **Tumor suppressor protein 53** (TP53) induced apoptosis involves the transcriptional induction of redox proteins, generation of reactive oxygen species, and oxidative degradation of mitochondrial components that results in cell death. TP53 was shown to transcriptionally regulate proapoptotic proteins like Bax and NOXA (Yamada et al., 2002).
- **NF-kB** expression is implicated in the survival of living cells. NF-kB family contains five proteins namely c-Rel, RelA, RelB, p50/p105, and p52/p100. NF-kB as a homo or hetero dimers bind to the kB sites on their target DNA and regulate their expression (Barkett and Gilmore, 1999). NF-kB is activated by various stimuli like pathogens, mitogens, proinflammatory cytokines, etc. It plays a major role in immune responses and affects the expression of genes c-IAP-1 and c-IAP-2, Fas ligand, c-myc, p53, etc. involved in apoptosis (Zhang and Ghosh, 2001). Two TNF receptors TNFRSF8 and TNFRSF9 were shown to promote apoptosis, former activating, and latter inactivating NF-kB expression (Wang et al., 2008).

## Apoptosis and *mycobacterium tuberculosis*

*M. tuberculosis* infections with virulent strains have been reported to inhibit macrophage apoptosis (Behar et al., 2010). Varied mechanisms of apoptotic suppression have been reported in *M. tuberculosis* infections (Table 1) unraveling the tactics of this pathogen to generate a protective niche inside the host. Among the various cell death modalities described above, only three apoptotic responses were documented in *M. tuberculosis* infection namely apoptosis (*nuoG*, *SecA2*, *pknE*, *lpqH*, *esxA* (ESAT-6), PE_PGRS33, *pstS-1*, Rv3654c, and Rv3655c), pyroptosis (*zmp1*, Rv3364c), and autophagy (*eis*) (Hinchey et al., 2007; Velmurugan et al., 2007; Jayakumar et al., 2008; Master et al., 2008; Sanchez et al., 2009, 2012; Danelishvili et al., 2010, 2012; Shin et al., 2010).

**Table 1:** **Apoptotic mechanisms in the pathogenesis of *M. tuberculosis***.

**S.no**	**Mechanisms of apoptosis**	**Year**	**References**
1	Treatment of macrophages post-infection with exogenous ATP reduces viability	1994	Molloy et al., 1994
2	Extrinsic apoptosis	1997	Keane et al., 1997
3	Virulent strains induce IL-10-dependent sTNFR2 forming inactive TNF-α-TNFR2 complex	1998	Fratazzi et al., 1999
4	Granulysin and perforin reduce the viability of *M. tuberculosis*	1998	Stenger et al., 1998
5	Treatment of Fas ligand post-infection reduces the viability	1998	Oddo et al., 1998
6	Degree of apoptosis is strain-dependent	2000	Keane et al., 2000
7	ManLam prevents apoptosis by altering Ca^2+^ levels	2000	Rojas et al., 2000
8	*M. tuberculosis* apoptosis down regulates CD14	2000	Santucci et al., 2000
9	Apoptosis of avirulent strains dependent on group IV cytosolic phospholipase A_2_ and TNF-α	2001	Duan et al., 2001
10	Reduced viability using exogenous ATP is executed using P2X7 receptor	2001	Fairbairn et al., 2001
11	Anti-apoptotic Mcl-1expression by virulent strains decreases apoptosis	2003	Sly et al., 2003
12	Detour pathway of antigen presentation	2003	Schaible et al., 2003
13	19 kDa lipoprotein induces apoptosis by TLR2 signaling	2003	Lopez et al., 2003
14	Virulent strains induce necrosis	2006	Park et al., 2006
15	Methyl glyoxal plays role in apoptosis	2006	Rachman et al., 2006
16	TLR-2-mediated activation of NF-kB and c-FLIP protects infected cells from FasL-induced apoptosis	2006	Loeuillet et al., 2006
17	PE_PGRS33 induces TNF-α secretion using TLR-2 signaling and genetic alterations in PE_PGRS33 decreases TNF-α secretion	2006	Basu et al., 2007
18	High MOI induces TNF-α independent apoptosis leading to mycobacterial spread	2007	Lee et al., 2006
19	Higher MOI leads to caspase independent apoptosis involving both mitochondria and lysosymes	2007	O'Sullivan et al., 2007
20	ESAT-6 induces apoptosis	2007	Derrick and Morris, 2007
21	Bystander apoptosis elicited by avirulent strains are independent of TNF-α,Fas,TRAIL, TGF-β, TLR2, and MyD88	2008	Kelly et al., 2008
22	Virulent strains prevents apoptotic envelope formation leading to necrosis	2008	Gan et al., 2008
23	Virulent strains produce more lipoxinA_4_ promoting necrosis and avirulent strain induces PGE_2_ that prevents necrosis	2008	Chen et al., 2008
24	Formation of NETs unable to kill *M. tuberculosis*	2008	Ramos-Kichik et al., 2009
25	Prevents pyroptosis using *zmp*1 by inhibiting inflammasome formation required for IL-1β secretion	2008	Master et al., 2008
26	pstS1 induces TNF-α, FasL,Fas TNFR1, TNFR2, and TLR-2 mediated apoptosis	2008	Sanchez et al., 2009
27	TNF-α-mediated caspase-8 apoptosis by p38MAPK, ASK-1, and FLIP_S_ degradation	2009	Kundu et al., 2009
28	Virulent strains inhibit plasma membrane repair promoting necrosis	2009	Divangahi et al., 2009
29	Neutrophil activation leads to ectososme release	2010	Gonzalez-Cano et al., 2010
30	*nuoG* neutralize NOX2 derived ROS inhibiting extrinsic apoptosis	2010	Miller et al., 2010
31	Rv3654c and Rv3655c genes prevent extrinsic apoptosis	2010	Danelishvili et al., 2010
32	*eis* is involved in suppressing autophagy in a redox dependent JNK activation	2010	Shin et al., 2010
33	Higher MOI induces host cell lipolysis and PHOPR kinase plays a role in this response	2011	Divangahi et al., 2009
34	PE_PGRS33 interacts with host mitochondria and probably involved in primary necrosis	2011	Cadieux et al., 2011
35	Dendritic cells undergo caspase independent apoptosis	2011	Ryan et al., 2011
36	ROS mediated necrosis as a survival strategy in neutrophils	2012	Corleis et al., 2012
37	ESAT-6 induced apoptosis is regulated by BAT3	2012	Grover and Izzo, 2012
38	Rv3364c prevents pyroptosis by inhibiting cathepsinG	2012	Danelishvili et al., 2012
39	*pknE* inhibits various modes of apoptosis in response to nitric oxide stress of the macrophages	2012	Kumar and Narayanan, 2012
40	nuoG mutant reveals decreased neutrophil apoptosis reduces CD4 T cell activation	2012	Blomgran et al., 2012
41	Virulence determines cytotoxicity whereas strain characteristics determine the mode of cell death	2012	Butler et al., 2012
42	ESAT-6 is involved in inhibiting autophagy	2012	Romagnoli et al., 2012
43	sigH or its regulated genes suppresses apoptosis, modulates innate immune responses, and reduces chemotaxis	2012	Dutta et al., 2012
44	Infection with avirulent mycobacteria induces mitochondrial exhaustion while virulent promotes mitochondrial function thereby increasing ATP synthesis	2012	Jamwal et al., 2013
45	LpqH induces both extrinsic and intrinsic apoptosis	2012	Sanchez et al., 2012
46	Virulent Mycobacterial strains induce apoptosis by ESX-1 system and colonize new cells	2013	Aguilo et al., 2013
47	Validation of burst size hypothesis in *in vivo* model	2013	Repasy et al., 2013
48	*pknE* involved in the copathogenesis of HIV/TB coinfection	2014	Parandhaman et al., 2014

This table illustrates varied apoptotic mechanisms identified in the pathogenesis of M. tuberculosis. The abbreviations MOI denote multiplicity of infection, ManLam, mannosylated lipoarabinomannan; PGE_2_, prostaglandinE2; ROS, reactive oxygen species; ATP, adenosine tri phosphate.

## Serine/threonine protein kinases (STPK)

Two component signaling systems were considered as the standalone mechanism of signaling in prokaryotes in response to environmental cues. However with the availability of various molecular techniques serine, threonine, and tyrosine mediated phosphorylation events unique to eukaryotes were documented in pathogenic prokaryotes like *M. tuberculosis*, *Streptococcus* species, Staphylococcus spp, *Pseudomonas* spp, etc. (Chao et al., 2009; Chakraborti et al., 2011). Among the 11 STPKs that *M. tuberculosis* encodes, only five of them *pknE*, *pknG*, *pknH*, *pknI,* and *pknK* were reported to support intracellular survival (Walburger et al., 2004; Papavinasasundaram et al., 2005; Jayakumar et al., 2008; Gopalaswamy et al., 2009; Malhotra et al., 2010). Our data for the first time proved that PknE was the only STPK to inhibit apoptosis (Jayakumar et al., 2008).

## pknE in innate immunity

The function of *pknE* was established from our studies using the deletion mutant Δ*pknE* generated using specialized transduction. Deletion of *pknE* had reduced intracellular survival, increased apoptosis, and reduced proinflammatory responses (Jayakumar et al., 2008). Subsequent molecular pathogenesis studies revealed that the deletion of *pknE* promotes macrophage cell death dependent on intrinsic pathway of apoptosis, TP53, and *Arg2*. This apoptosis was independent of TNF-α, iNOS, Akt, *Arg1,* and pro-inflammatory cytokines (Kumar and Narayanan, 2012). *M. tuberculosis* encounters reactive nitrogen and oxygen intermediates inside the macrophages as one among the host defenses. Characterization of the promoter of the *pknE* gene showed its elevated expression during nitric oxide (NO) stress (Jayakumar et al., 2008). Macrophage experiments performed using NO donor sodium nitroprusside to mimic the host microbicidal activity confirmed that, *pknE* in response to NO stress suppresses innate immune responses (Kumar and Narayanan, 2012). *In vitro* studies carried with the deletion mutant showed defective growth in pH 7.0 and lysozyme (a cell wall-damaging agent) with better survival in pH 5.5, SDS (surfactant stress), and kanamycin (a second-line anti-tuberculosis drug). Δ*pknE* was reduced in cell size during growth in liquid media and exhibited hypervirulence in a guinea pig model of infection (Kumar et al., 2012). The data from the *in vitro* studies highlighted the role of *pknE* in adaptive responses of *M. tuberculosis*. Recently we reported that, deletion of *pknE* results in defective phosphorylation kinetics of MAPKs (p38MAPK, Erk½, and SAPK/JNK) and their transcription factors ATF-2 and c-JUN. Deletion of *pknE* also revealed crosstalks in the host macrophages where Erk½ signaling was found to be influenced by SAPK/JNK and p38 pathways independently. Modulations in intra cellular signaling altered the expression of coreceptors CCR5 and CXCR4 in macrophages infected with the deletion mutant of *pknE* that were authenticated using HIV tropic strains (Parandhaman et al., 2014). For the first time, our data showed that difference in apoptosis and intracellular signaling events, and the virulence capacity of the *M. tuberculosis* strain could influence the copathogenesis of HIV infection (Parandhaman et al., 2014). Collectively the reports show that *pknE* has a role suppression of innate immunity and help *M. tuberculosis* to adapt to the different environmental condition that it encounters.

## Conclusion

Molecular techniques have revolutionized our understanding of pathogenic organisms and their interactions with the immune system. Pathogenic organisms have evolved host mimicking properties and utilize the host responses for their own survival and propagation. This review has addressed the various mechanisms of cell death that is vital for initiating an innate and adaptive immunity against the invading pathogen. As novel cell death paradigms evolve, it adds to the complexity of how temporally and spatially the immune system coordinates these responses. Most of the cell death models described here disrupt the energy source of the cell, mitochondria indicating whether these paradigms are interconnected response of a single biochemical event and this still remains a puzzle. Adding complexity to this conundrum is that, pathogenic organisms like *M. tuberculosis* is able to inhibit the various apoptotic models that were discovered so far. This arise the question whether *M. tuberculosis* by educating itself avoids cell death or has antigens that are poor inducers of cell death and that await further studies.

### Conflict of interest statement

The authors declare that the research was conducted in the absence of any commercial or financial relationships that could be construed as a potential conflict of interest.
